# Alpha-Enolase Protects Hepatocyte Against Heat Stress Through Focal Adhesion Kinase-Mediated Phosphatidylinositol 3-Kinase/Akt Pathway

**DOI:** 10.3389/fgene.2021.693780

**Published:** 2021-07-19

**Authors:** Tao Zeng, Yongqing Cao, Tiantian Gu, Li Chen, Yong Tian, Guoqin Li, Junda Shen, Zhenrong Tao, Lizhi Lu

**Affiliations:** ^1^Institute of Animal Husbandry and Veterinary Medicine, Zhejiang Academy of Agricultural Sciences, Hangzhou, China; ^2^Key Laboratory of Information Traceability for Agricultural Products, Ministry of Agriculture of China, Hangzhou, China; ^3^Jiangsu Key Laboratory for Animal Genetics, Breeding and Molecular Design, Yangzhou University, Yangzhou, China

**Keywords:** ENO1, Hsp70, glycolysis, cell viability, FAK-PI3K/AKT

## Abstract

Accumulating pieces of evidence showed that α-enolase (ENO1) is a multifunctional protein that plays a crucial role in a variety of pathophysiological processes. In our previous study, differential expression of ENO1 was observed in different heat-tolerance duck breeds. Here, we examined *in vitro* expression level of ENO1 in hepatocytes against heat stress. The mechanisms of ENO1 on cell glycolysis, growth, and its potential regulatory pathways were also analyzed. The results showed that ENO1 expression in messenger RNA and protein levels were both greatly increased in heat-treated cells compared with non-treated cells. ENO1-overexpressed cells significantly elevated cell viability and glycolysis levels. It was further shown that stably upregulated ENO1 activated focal adhesion kinase-phosphatidylinositol 3-kinase/Akt and its downstream signals. In addition, the interaction between ENO1 and 70-kDa heat shock protein was detected using co-immunoprecipitation. Our research suggests that ENO1 may interact with 70-kDa heat shock protein to protect hepatocyte against heat stress through focal adhesion kinase-mediated phosphatidylinositol 3-kinase/Akt pathway.

## Introduction

Heat stress is defined as external factors acting on an animal to induce a body temperature increase that arouses a series of physiological responses (Dikmen and Hansen, 2009; Te Pas et al., 2019). It is one of the most common and inevitable etiological phenomena in livestock and poultry breeds. Poultry is more vulnerable to heat stress due to its special physiological structure, such as no sweat glands and thick feathers.

Decreases in food consumption, feeding efficiency, growth rate, and survival ability were found in some studies when animals were under heat stress conditions (van der Hel et al., 1992; Geraert et al., 1996). In addition, meat production, quality (including color, water holding capacity, and share force value), egg production rates, and quality (including egg weight, shell weight, shell thickness, and specific gravity) were also affected (Mashaly et al., 2004; Bayhan et al., 2013; Hashizawa et al., 2013).

Cells can exert intrinsic defense mechanisms against heat stress actively. α-Enolase (ENO1), which is found in almost all human tissues, was originally described as an enzyme related to the glycolytic pathway (Marangos et al., 1978; Díaz-Ramos et al., 2012). With the exception of its glycolytic function, an increasing number of studies have shown that ENO1 is a multifunctional protein involved in various pathophysiological and biological processes based on its cellular localization (Martindale and Holbrook, 2002). ENO1-overexpressed cells were observed in human non-small cell lung cancer cell lines, which can facilitate cell glycolysis, migration, proliferation, tumorigenesis, and invasion (Fu et al., 2015). Another study demonstrated that ENO1 interacts with constitutive 70-kDa heat shock protein (HSP70) and protects against oxidative stress in rat cardiomyocytes *via* enhancing glycolysis pathway and energy metabolism levels (Luo et al., 2011). Wang et al. (2012) showed that ENO1 expression was presented as a bell-shaped regulation responding to temperature change. Iida and Yahara (1985) demonstrated that the ENO1 gene could encode HSP48 in *Saccharomyces cerevisiae*. HSP48 was durably induced when cells were induced to enter the dormancy period, and those cells were much more resistant to heat shock than others. In our previous study, we found that ENO1 showed different expression patterns in two duck species against heat stress (Zeng et al., 2013). Furthermore, we compared ENO1 gene expression levels in liver tissues of two duck breeds after heat stress. The results showed that ENO1 expression levels were upregulated in the thermal tolerance breed (Zeng et al., 2014). We also found that ENO1 and HSP70 showed a collaborative expression trend using proteomics analysis (Zeng et al., 2017).

In the present study, we analyzed the expression of ENO1 in hepatocytes against heat stress, as well as its effects on cell glycolysis, growth, and modulatory mechanisms in focal adhesion kinase (FAK)-mediated phosphatidylinositol 3-kinase (PI3K)/Akt pathway (Wei and Vander Heide, 2008; Park et al., 2016; He et al., 2019; Xu et al., 2019). In addition, we also used co-immunoprecipitation (co-IP) to examine the interaction between ENO1 and HSP70 against heat stress.

## Materials and Methods

### Cell Culture and Treatment

Chicken embryo hepatocytes were purchased from Otwo Biotech Inc. (Shenzhen, China). It is the primary cell that can be passaged for 10–15 (P10–15), with one passage for approximately 5 days. Cells were cryopreserved by 92% complete medium and 8% dimethyl sulfoxide. After resuscitation, adherent chicken embryo hepatocytes were delivered in a T25 cell culture flask. The cells were adaptively cultivated in Roswell Park Memorial Institute 1640 (HyClone, Logan, United States) supplemented with 10% fetal bovine serum (HyClone, Logan, United States) at 37°C. After adaptive culture, heat-stressed cells were cultivated in high glucose Dulbecco’s modified Eagle’s medium (HyClone, Logan, United States) supplemented with 10% fetal bovine serum at 41°C for 0, 6, 12, 24, or 36 h, whereas control cells were cultivated at 37°C for the same time.

### Plasmid Construction, Small Interfering RNAs, and Cell Transfection

A recombined plasmid carrying full-length chicken ENO1 (Tanaka et al., 1995) was synthesized by General Biosystems Anhui Co., Ltd. (Chuzhou, China). After digesting with *Xba*I-*Bam*HI, the recombined plasmid was combined with the lentiviral vector pCDH-CMV-MCS-EF1-copGFP (General Biosystems, Chuzhou, China). Next, the vector carrying full-length ENO1 or empty vector (for control) was transfected into chicken hepatocytes using Lipofectamine^TM^ 2000 Transfection Reagent (Thermo Fisher Scientific, Waltham, United States). The transfection procedure was conducted using the manufacturer’s protocol.

Three small interfering RNAs (siRNAs) for ENO1 (RNA-1, RNA-2, and RNA-3) were designed and synthesized by GenePharma Co., Ltd. (Shanghai, China). A negative control (NC) siRNA was also designed and synthesized by the company. The siRNAs and NC were transfected into chicken hepatocytes using Lipofectamine^®^ RNAiMAX Reagent (Thermo Fisher Scientific, United States). The transfection procedure was performed using the manufacturer’s protocol.

### Cell Viability 3-(4, 5-Dimethylthazol-2-Yl)-2,5-Diphenyl Tetrazolium Bromide Assay

Chicken hepatocytes were inoculated in 96-well plates (12,000 cells per well) to attach for 24 h at 37°C. After treating the heat and control groups, the cells in each well were treated with 20-μl 3-(4,5-dimethylthazol-2-yl)-2,5-diphenyl tetrazolium bromide (MTT) (5 mg/ml) (Sigma, San Francisco, United States) and incubated for 4 h at 37°C. After incubation, 150-μl dimethyl sulfoxide (Sangon Biotech, Shanghai, China) was added to each well before shaking for 10 min to dissolve crystals. The absorbance value was analyzed using a Multiscan Spectrum (PerkinElmer, United States) at 490 nm.

### Quantitative Reverse Transcription-Polymerase Chain Reaction

Total RNA was extracted from chicken hepatocytes using TaKaRa MiniBEST Universal RNA Extraction Kit (Takara, Dalian, China). RNA quality was assessed after DNA removal, and RNA (0.7413 μg) was reverse transcribed. The quantitative reverse transcription-polymerase chain reaction (qRT-PCR) system contained 1-μl complementary DNA, 1-μl sense primer, 1-μl antisense primer, 10-μl 2 × mix, and 7-μl water. Specific ENO1, HSP70, and glyceraldehyde 3-phosphate dehydrogenase (reference gene) primers are presented in Table 1. PCR conditions began at 95°C for 10 min followe3d by 40 cycles of 95°C for 10 s and 60°C for 15 s. Amplification product specificity was assessed using a melting curve. Each sample was amplified using three technical replicates, and independent experiments were performed in triplicate. Data were normalized using the 2^–Δ Δ^ ^*CT*^ method.

**TABLE 1:** Primer sequences for all genes in this study.

Gene		Sequence (5′–3′)	Accession no.	Size
HSP70	Sense	GCCCAGAACATCATCCCA	NM_001006685.1	144 bp
	Antisense	CGGCAGGTCAGGTCAACA		
ENO1	Sense	TGGATGGAACGGAGAACA	NM_205120.1	159 bp
	Antisense	AAGCAGGAACAGGCAGAA		
LDHA	Sense	TCTGGAGCGGAGTGAATG	NM_205284.1	106 bp
	Antisense	ACCACCTGCTTGTGAACC		
FAK	Sense	ATGTTATTGGTCGGATTG	NM_205435.1	113 bp
	Antisense	GGGTCGTCTACTAGGGTC		
PI3K	Sense	TAGCGGTGCTCGTGTAAG	NM_001004410.1	165 bp
	Antisense	GAGAAATCCTGTGGGTGG		
Akt	Sense	GAGTATGCTAACGGAGGG	NM_205055.1	258 bp
	Antisense	TGGAGTGCCACAGAAAGT		
GAPDH	Sense	GGCTCCACTCGTATTCCT	NM_204305.1	137 bp
	Antisense	TTCAGACTTGTCTCCCATG		
NC siRNA	Sense	UUCUCCGAACGUGUCACGUTT		
	Antisense	ACGUGACACGUUCGGAGAATT		
siRNA-1	Sense	GAAGTTCACTGCCAGTGTTTT		
	Antisense	AACACTGGCAGTGAACTTCTT		
siRNA-2	Sense	TGCTCCTTAAGGTCAACCATT		
	Antisense	TGGTTGACCTTAAGGAGCATT		
SiRNA-3	Sense	GAACCCTCGTATCAACTAATT		
	Antisense	TTAGTTGATACGAGGGTTCTT		

### Western Blot

Chicken hepatocyte in each well was treated with 150–250-μl radioimmunoprecipitation assay lysis buffer (Beyotiome, Shanghai, China). Cell sediment and lysate were centrifuged at 12,000 rpm and 4°C for 3–5 min. The supernatant was collected to detect total protein using a BCA Protein Assay Kit (Beyotiome, Shanghai, China). The protein contained in cell lysates was resolved using a 12% sodium dodecyl sulfate–polyacrylamide gel electrophoresis gel before transferring to a polyvinylidene fluoride (PVDF) membrane. The membranes were washed with Tris-buffered saline + Tween and blocked with 5% milk/phosphate-buffered saline with Tween (PBST) for 1 h. The primary Abs, diluted with 1% bovine serum albumin/PBST, were closely incubated with the PVDF membrane overnight at 4°C. After washing, the membrane was placed into horseradish peroxidase-labeled second Abs diluted with 5% milk/PBST and incubated for 1 h at room temperature. The PVDF membrane was visualized using enhanced luminol reagent and oxidizing reagent and detected using the gel imaging system (ChemiDoc^TM^ XRS+, Bio-Rad, Hercules, United States).

The primary antibodies and dilutions used in this study included the ENO1 antibody (1:1,000, Abcam, Cambridge, United Kingdom), HSP70 antibody (1:1,000, Abcam, Cambridge, United Kingdom), lactate dehydrogenase A (LDHA) antibody (1:1,000, Chicago, Proteintech, United States), FAK antibody (1:1,000, Proteintech, United States), PI3K antibody (1:1,000, Proteintech, Chicago, United States), Akt antibody (1:1,000, Proteintech, Chicago, United States), and glyceraldehyde 3-phosphate dehydrogenase antibody (1:3,000, AtaGenix, Wuhan, China).

### Lactic Acid Assay

The cell lysate supernatant was collected to detect lactic acid content using the Lactic Acid Assay Kit (Nanjing Jiancheng Bioengineering Institute, Nanjing, China) according to the manufacturer’s guidance. Absorbance values of color material were measured using a visible spectrophotometer (Shanghai Metash Instruments Co., Ltd., Shanghai, China) at 630 nm. The cellular lactic acid content was linearly related to the absorbance value.

### Co-immunoprecipitation

Cells in each well were treated with cell lysis buffer for Western blotting and immunoprecipitation (Beyotiome, Shanghai, China). Lysis proceeded for 30 min on ice before centrifugation at 12,000 rpm at 4°C for 5 min. The supernatant was collected and added to 100-μl protein A + G agarose beads (Beyotiome, Shanghai, China), rotating for 1 h to remove non-specific binding proteins. After diluting, ENO1 or HSP70 (1:1,000) antibodies [normal immunoglobulin G (IgG) (1:3,000) for the control group] were combined with protein A + G agarose beads. The cell lysate was added to protein A + G agarose beads containing conjugated Abs or normal IgG. The mixture was rotated overnight at 4°C to combine protein with Abs. The sample was used to perform Western blotting after centrifuging and washing. The results were detected using the same method as Western blotting.

### Statistical Analysis

All data were independently repeated at least three times and presented as mean ± standard error of the mean. Statistical significance (*p* < 0.05) for each variable was analyzed using unpaired *t-*test or variance (analysis of variance) in SPSS statistical software (version 20.0.0 for Windows, IBM Corp., Armonk, United States).

## Results

### Construction of Heat Stress Model

**FIGURE 1:**
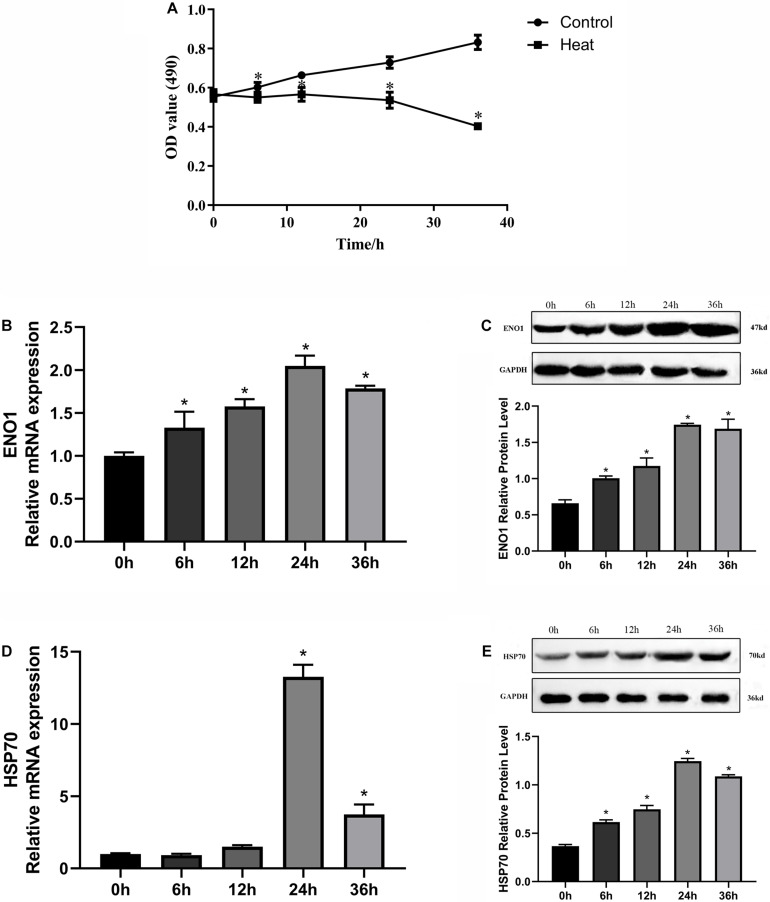
Effects of heat stress on cell viability, ENO1 and HSP70 expression in chicken hepatocytes. **(A)** Cell viability gradually decreased after treatment at 41°C for 0, 6, 12, 24, and 36 h based on an MTT assay. **(B)** ENO1 mRNA and **(C)** protein levels after treatment at 41°C for different times based on qRT-PCR and Western blotting, respectively. **(D)** mRNA and **(E)** protein levels of HSP70 after treatment at 41°C for different periods. Data were replicated three times. GAPDH served as a loading control. Bars show mean ± SE (^∗^*p* < 0.05).

To construct an appropriate heat stress model *in vitro* and research gene expression that could be related to heat stress, we detected the cell viability and ENO1 and HSP70 expression levels in chicken hepatocytes after treatment at 41°C (37°C for control) for 0, 6, 12, 24, and 36 h. The cell viability was determined using MTT assays. The cell viability with treatment for 6, 12, 24, and 36 h was significantly decreased compared with control (*p* < 0.05, Figure 1A). The messenger RNA (mRNA) and protein levels of ENO1 were determined using qRT-PCR and Western blotting, respectively. The results showed that ENO1 mRNA levels increased compared with the control group and then reached the highest levels with treatment for 24 h (Figure 1B). The ENO1 protein level presented the same trend with its mRNA level along with heat stress time changing (Figure 1C). Meanwhile, HSP70 mRNA and protein levels also increased and followed a similar trend as ENO1 (Figures 1D,E). With comprehensively considering, the duration of heat treatment was set as 24 h in subsequent experiments.

### Construction of α-Enolase Upregulation and Downregulation Cell Lines

**FIGURE 2:**
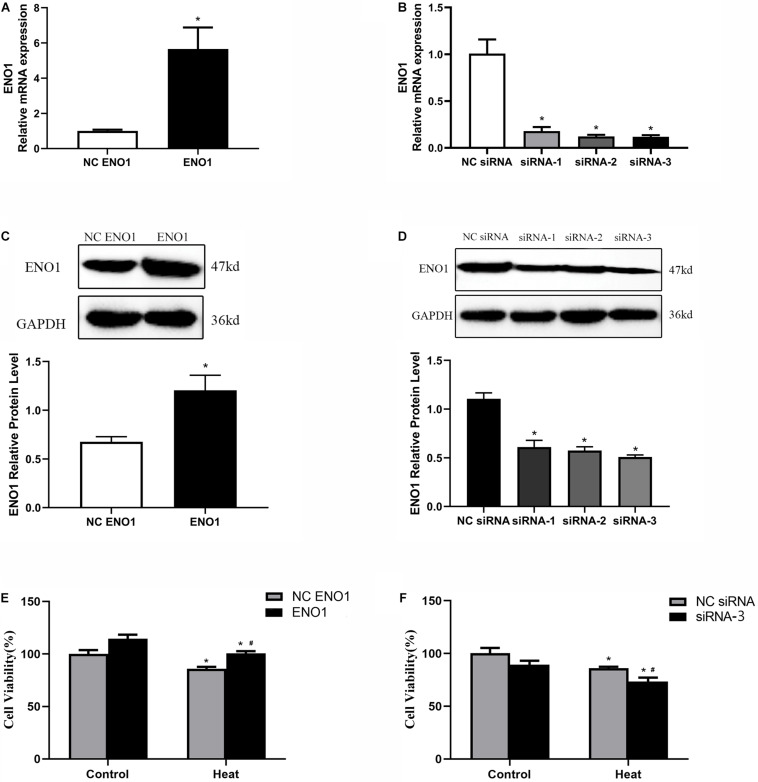
Construction of ENO1 upregulation and downregulation cell lines. ENO1 expression increased *via* stable transfection of full-length ENO1 based on **(A)** qRT-PCR and **(C)** Western blotting. ENO1 expression decreased *via* transient transfection of siRNA1, siRNA2, and siRNA3 based on **(B)** qRT-PCR and **(D)** Western blotting. **(E)** Cell viability increased in ENO1-overexpressed cells. **(F)** Cell viability decreased in ENO1-suppressed cells. Data were replicated three times. GAPDH served as a loading control. Bars show mean ± SE; ^∗^*p* < 0.05 vs. control group, ^#^*p* < 0.05 vs. NC ENO1 or NC siRNA + heat group.

To further study the effects of ENO1 expression levels on chicken hepatocytes under heat stress, we constructed cell lines of upregulated and downregulated ENO1 using cell transfection with lentiviral vector pCDH-CMV-MCS-EF1-copGFP, which carried full-length chicken ENO1 and empty vector (NC ENO1) and three siRNAs for ENO1 (siRNA-1, siRNA-2, and siRNA-3) and negative control siRNA (NC siRNA). The ENO1 mRNA and protein levels were detected using qRT-PCR and Western blotting, respectively. As shown in Figures 2A,C, compared with empty vector, ENO1 mRNA and protein levels were significantly increased when infected with full-length chicken ENO1. On the contrary, ENO1 mRNA and protein levels were significantly decreased in cells infected with siRNA-3 (*p* < 0.05, Figures 2B,D). To evaluate whether there is a correlation between ENO1 and cell viability in chicken cells under heat conditions, we measured the cell viability in ENO1 overexpression and deficiency cells. The MTT assay showed that ENO1 overexpression significantly elevated the cell viability compared with the NC group (*p* < 0.05, Figure 2E). Meanwhile, suppressed ENO1 significantly decreased the cell viability (*p* < 0.05, Figure 2F). These results suggested that ENO1 was involved in cell viability changes resulting from heat stress.

### Glycolysis Levels in α-Enolase-Overexpressing and α-Enolase-Suppressed Cells

**FIGURE 3:**
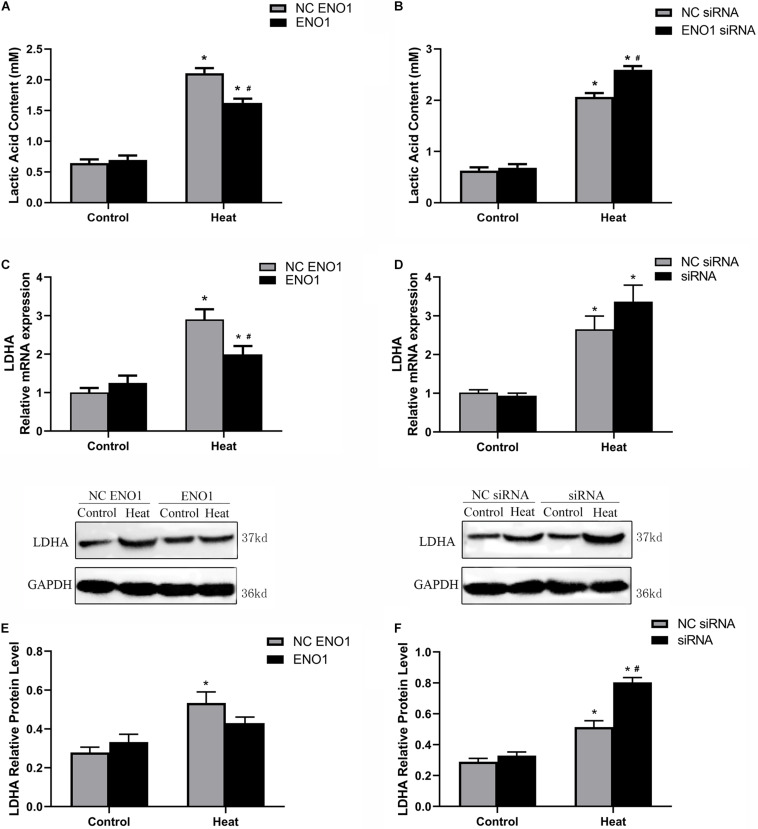
Lactic acid content and lactate dehydrogenase A (LDHA) expression level in ENO1-overexpressed and ENO1-suppressed cells. **(A)** Lactic acid content decreased in ENO1-overexpressed cells based on Lactic Acid Assay Kit. **(B)** Lactic acid content concentration increased in ENO1-suppressed cells. LDHA expression levels in ENO1-overexpressed cells based on **(C)** qRT-PCR and **(E)** Western blotting. LDHA expression levels increased in ENO1-suppressed cells based on **(D)** qRT-PCR and **(F)** Western blotting. Data were replicated three times. GAPDH served as a loading control. Bars show mean ± SE; ^∗^*p* < 0.05 vs. control group, ^#^*p* < 0.05 vs. NC ENO1 or NC siRNA + heat group.

To assess the effect of ENO1 expression on glycolysis, we first determined the concentration of LDHA using the Lactic Acid Assay Kit in ENO1-overexpressed and ENO1-suppressed chicken hepatocytes. We observed that ENO1 overexpression significantly reduced heat stress-induced lactic acid release (*p* < 0.05, Figure 3A), whereas the lactic acid concentration further remarkably increased in ENO1-suppressed cells (*p* < 0.05, Figure 3B). Furthermore, we examined the LDHA mRNA and protein levels using qRT-PCR and Western blotting, respectively (Figures 3C–F). In the control group, the mRNA and the protein levels of LHDA showed slight differences with siRNA but did not reach the significant level, which means that ENO1 has no significant effect on the expression of LDHA. The effect of ENO1 expression on LDHA mRNA and protein expression was proportional to its concentration (Figures 3C–F). However, no significant difference was detected for the protein expression of LDHA in ENO1-overexpressed cells (*p* > 0.05, Figure 3E). The results suggested that the glycolysis level was enhanced in chicken hepatocytes under heat stress, and ENO1 expression influenced the LDHA expression and concentration.

### Confirmation of Interaction Between α-Enolase and 70-kDa Heat Shock Protein

**FIGURE 4:**
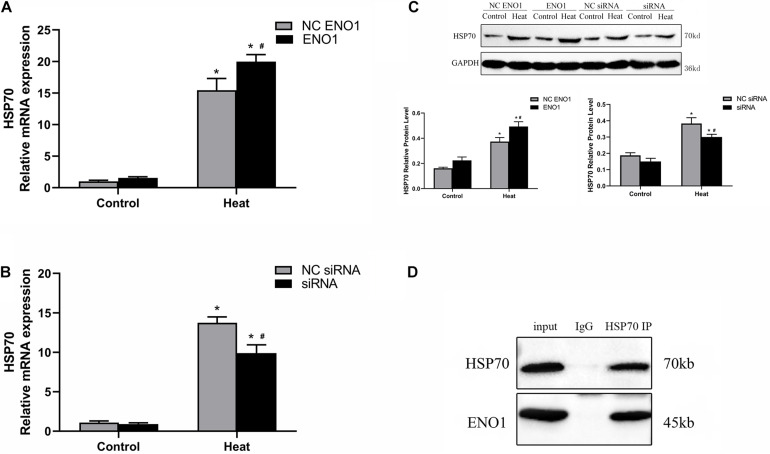
Confirmation of interaction between ENO1 and HSP70. **(A)** HSP70 mRNA level increased in ENO1-overexpressed cells. **(B)** HSP70 mRNA level decreased in ENO1-suppressed cells. **(C)** HSP70 protein level in ENO1-overexpressed or ENO1-suppressed cells. **(D)** Co-IP of ENO1 and HSP70 from chicken liver cell lysates using antibodies against HSP70. Chicken hepatocytes lysates based on Western blotting for positive control and non-specific rabbit IgG for negative control after heat treatment. Input means control panel. Data were replicated three times. GAPDH served as a loading control. Bars show mean ± SE; ^∗^*p* < 0.05 vs. control group, ^#^*p* < 0.05 vs. NC ENO1 or NC siRNA + heat group.

Because the expression tendency of HSP70 was similar to ENO1 over different lengths of time, it is reasonable to hypothesize that HSP70 and ENO1 interact. According to this conjecture, we determined the HSP70 mRNA and protein levels in ENO1-overexpressed/suppressed chicken hepatocytes, respectively. We also conducted co-IP using antibodies against HSP70 and non-specific rabbit IgG for control. The results showed that HSP70 mRNA and protein levels increased in ENO1-overexpressed chicken hepatocytes (Figures 4A,C). Similarly, HSP70 mRNA and protein levels decreased in ENO1-suppressed chicken hepatocytes (Figures 4B,C). The co-IP results showed that both ENO1 and HSP70 were detected in co-IP precipitates in cell lysate with the anti-HSP70 antibody (Figure 4D). The results hinted that ENO1 and HSP70 interact.

### Effects of α-Enolase Expression on Signal Factors, Focal Adhesion Kinase, Phosphatidylinositol 3-Kinase, and Akt

**FIGURE 5:**
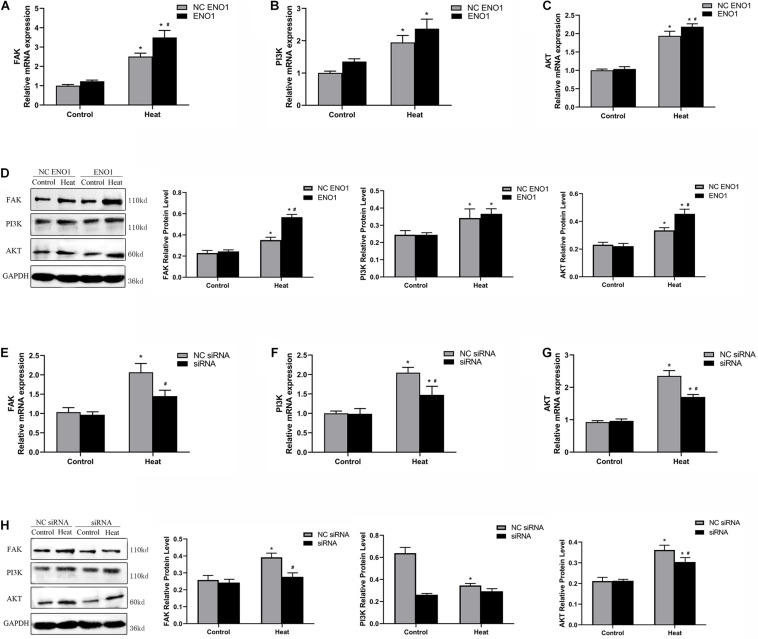
Effects of ENO1 expression on signal factors, FAK, PI3K, and Akt. **(A)** FAK, **(B)** PI3K, and **(C)** Akt mRNA levels in ENO1-overexpressed cells. **(D)** FAK, PI3K, and Akt protein levels in ENO1-overexpressed cells. **(E)** FAK, **(F)** PI3K, and **(G)** Akt mRNA levels in ENO1-suppressed cells. **(H)** FAK, PI3K, and Akt protein levels in ENO1-suppressed cells. Data were replicated three times. GAPDH served as a loading control. Bars show mean ± SE; ^∗^*p* < 0.05 vs. control group, ^#^*p* < 0.05 vs. NC ENO1 or NC siRNA + heat group.

PI3K/Akt pathways have been reported to be a key signaling pathway involved in anti-heat stress (Zeng et al., 2014; He et al., 2019), and it is mediated by FAK. To further study the regulatory mechanism by which ENO1 elevates cell viability under heat stress, we detected FAK, PI3K, and Akt mRNA and protein levels in ENO1-overexpressed/suppressed cells. First, we observed that mRNA and protein levels for FAK, PI3K, and Akt were significantly increased in cells with heat treatment compared with the control group (*p* < 0.05, Figures 5A–D). Furthermore, FAK and Akt expression significantly increased in EON1-overexpressed cells compared with the control group under heat conditions (*p* < 0.05, Figures 5A,C). Although the PI3K increase did not reach a significant level, it also showed an upward trend (Figure 5B). On the contrary, the FAK, PI3K, and Akt mRNA levels were decreased in ENO1-suppressed cells, and the latter two of them reached significant levels of difference (*p* < 0.05, Figures 5E–G). The protein levels of these signal factors presented a similar expression trend as the mRNA (Figures 5D,H). Based on the results, we speculated that ENO1 is an upstream signal factor that can regulate the FAK-mediated PI3K/Akt pathway during heat stress.

### Effects of Angiotensin II and Wortmannin on α-Enolase, Focal Adhesion Kinase, Phosphatidylinositol 3-Kinase, Akt Expression Levels, Cell Viability, and Glycolysis Levels

**FIGURE 6:**
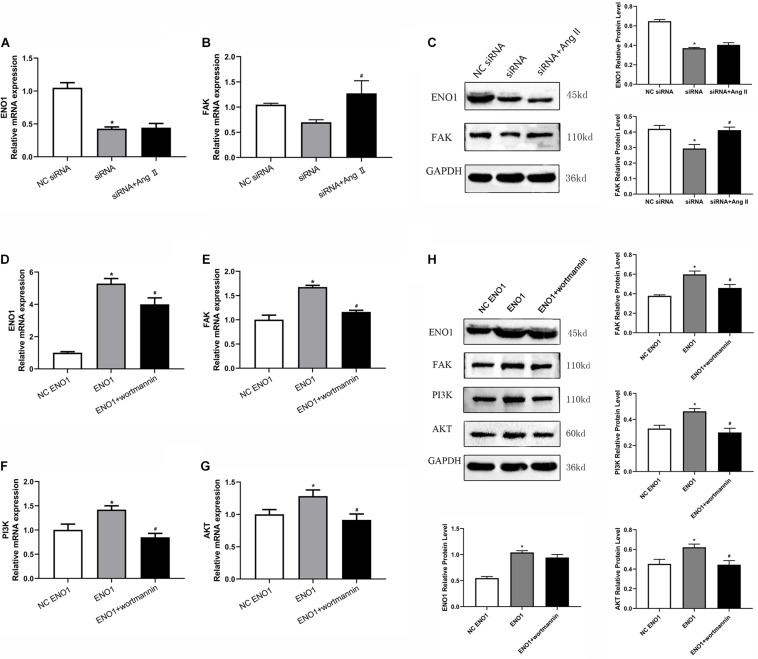
Effects of angiotensin II and wortmannin on ENO1, FAK, PI3K, and Akt expression levels. **(A)** There is no significant change of ENO1 mRNA level in ENO1-suppressed cells treat with Ang II during heat stress based on qRT-PCR. **(B)** FAK mRNA levels increased in ENO1-suppressed cells with Ang II treatment during heat stress. **(C)** Protein levels of ENO1 and FAK in ENO1-suppressed cells with Ang II treatment during heat stress. **(D)** ENO1, **(E)** FAK, **(F)** PI3K, and **(G)** Akt mRNA levels all decreased in ENO1-overexpressed cells with wortmannin treatment during heat stress. **(H)** Protein levels of ENO1, FAK, PI3K, and Akt in ENO1-overexpressed cells with wortmannin treatment during heat stress. Data were replicated three times. GAPDH served as a loading control. Bars show mean ± SE; ^∗^*p* < 0.05 vs. NC ENO1 or siRNA group, ^#^*p* < 0.05 vs. ENO1 or siRNA group.

Angiotensin (Ang) II is a FAK accelerant (Dong et al., 2013), whereas wortmannin is a PI3K/Akt inhibitor (Abliz et al., 2015). To further confirm ENO1’s regulatory mechanism, Ang II and wortmannin were treated in ENO1-overexpressed/suppressed cells. Results showed that FAK mRNA and protein levels both increased (Figures 6B,C), but there was no significant difference in ENO1 expression (*p* > 0.05, Figures 6A,C) after Ang II treatment in ENO1- suppressed cells during heat stress. The results suggested there is no feedback regulation from FAK to ENO1. The FAK, PI3K, and Akt mRNA and protein levels all decreased after wortmannin treatment in ENO1-overexpressed cells during heat stress. (*p* < 0.05, Figures 6D–H). We hypothesize that there is positive regulation between ENO1 and these signal factors, combined with the influence of ENO1 expression levels on FAK, PI3K, and Akt.

**FIGURE 7:**
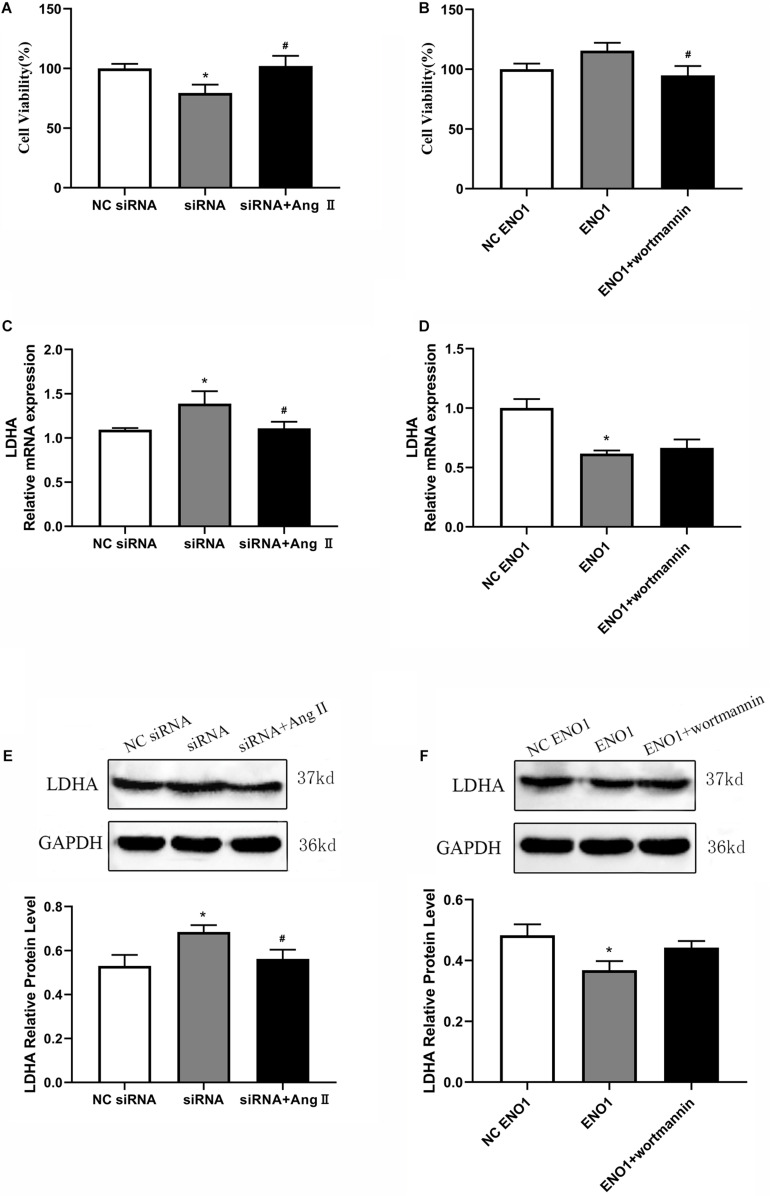
Effects of angiotensin II and wortmannin on cell viability and LDHA expression levels. **(A)** Cell viability increased in ENO1-suppressed cells with Ang II treatment during heat stress. **(B)** Cell viability decreased in ENO1-overexpressed cells with wortmannin treatment during heat stress. LDHA mRNA **(C)** and protein **(E)** levels in ENO1-suppressed cells with Ang II treatment during heat stress. No significant changes were found for LDHA mRNA **(D)** and protein levels **(F)** in ENO1-overexpressed cells with wortmannin treatment during heat stress. Data were replicated three times. GAPDH served as a loading control. Bars show mean ± SE; ^∗^*p* < 0.05 vs. NC ENO1 or siRNA group, ^#^*p* < 0.05 vs. siRNA + Ang II or ENO1 + wortmannin group.

Cell viability was increased in ENO1-suppressed cells with Ang II treatment during heat stress based on MTT assay (Figure 7A). Meanwhile, after treatment with wortmannin, cell viability was significantly decreased in ENO1-overexpressed cells (Figure 7B). We then detected the mRNA and protein levels of LDHA in ENO1-suppressed/overexpressed cells with Ang II and wortmannin treatment. The results showed that LDHA mRNA and protein levels decreased in ENO1-suppressed cells with Ang II treatment during heat stress (Figures 7C,E). However, no significant changes were found for LDHA mRNA and protein levels in ENO1-overexpressed cells with wortmannin treatment (Figures 7D,F).

## Discussion

Enolase, discovered by Lohman and Mayerhof in 1934, plays an important role in the glycolysis pathway that catalyzes 2-phosphoglycerate dehydration to phosphoenolpyruvate in the second half of the glycolytic pathway (Capello et al., 2011). ENO1 (2-phospho-D-glycerate hydrolyase), a 48-kDa protein primarily found in the cytoplasm (Feo et al., 2000), is one of three enolase isoforms (ENO1, β-enolase, and γ-enolase) (Marangos et al., 1978), which is widely expressed in many vertebrate organisms and can be found in various tissue types including liver (Pancholi, 2001). Previous studies have demonstrated that ENO1 played some significant role in protecting cells against adverse effects such as cancers (Tsai et al., 2010; Song et al., 2014; Fu et al., 2015; Yin et al., 2018), stresses (Aaronson et al., 1995; Lu et al., 2014), and other pathophysiological processes (Chen et al., 2014). In response to hypoxia stress, Aaronson et al. (1995) suggested that enolase contributes to vascular endothelial cells obtaining hypoxia tolerance. Another study showed that ENO1 interacts with constitutive HSP70 and protects against oxidative stress in rat cardiomyocytes *via* enhancing glycolysis pathway and energy metabolism levels (Luo et al., 2011). A report by Fu et al. (2015) indicated ENO1 was overexpressed in non-small cell lung cancer, promoting cell glycolysis, proliferation, migration, invasion, and tumorigenesis by activating the FAK-mediated PI3K/Akt pathway and further modulating their downstream signal molecules (Song et al., 2014). In our previous study, we detected several proteins, including ENO1, which showed different expression patterns in different heat-tolerant duck breeds (Zeng et al., 2013, 2017). These unexpected findings provided us a new insight into the role of ENO1 in acquiring thermal tolerance. Therefore, we constructed a heat stress model on the chicken hepatocytes *via* heat exposure for different time periods. Upregulated expression of ENO1 has been detected in heat stress cell compared with control groups. Further studies found that overexpressed ENO1 increased cell viability, reduced heat stress-induced LDHA release on chicken hepatocytes after heat stress. These data suggested that ENO1 was involved in modulating cell viability and glycolysis levels during heat stress.

HSP70 was shown to be a molecular chaperone that prevents inappropriate protein aggregation and mediates immature protein transport to the target organelles for final packaging, degradation, or repair (Kiang and Tsokos, 1998; Mayer and Bukau, 2005; Young, 2010). It was previously shown that HSP70 is involved in cell death and survival processes caused by heat stress *in vitro* (Pavlik et al., 2003). In addition, our previous studies suggested that HSP70 played an important role in protecting duck liver from heat stress (Zeng et al., 2014). To understand the relationship between HSP70 and ENO1 in the process of thermal stress, we determined the expression of HSP70 in ENO1-overexpressed/suppressed cells and conducted co-IP between them. The result showed that HSP70 expression also increased or decreased when we upregulated or downregulated ENO1 during heat stress, respectively. More importantly, the protective effect of ENO1 on the chicken hepatocytes against heat stress was partly associated with its interaction with HSP70. These results suggested that except for influencing glycolysis, ENO1 may have a role in enhanced chaperoning function *via* interacting with HSP70, and this will require further analyses.

PI3K is a lipid kinase that plays a crucial role in regulating cell apoptosis, senescence, cellular metabolism, and motility (Akinleye et al., 2013). PI3K transmits signals from the cell surface to the cytoplasm by generating secondary messengers that activate multiple effector kinase pathways (Cantley, 2002; Burris, 2013). Akt is a downstream signal factor of PI3K, which regulates the function of a variety of substrates involved in modulating cellular growth, cell survival, and cell cycle in tumorigenesis and cancer progression (Chen et al., 2001; Kandasamy and Srivastava, 2002; Yuan and Whang, 2002). Several studies have shown that PI3K/Akt pathway could be activated by cellular stress, such as ischemia, hypoglycemia, hypoxia, oxidative, and heat shock (Shaw et al., 1998; Ouyang et al., 2000; Ma et al., 2001; Sakurai et al., 2001; Martindale and Holbrook, 2002). The phenomenon of feedback regulation has been reported frequently in certain PI3K/Akt pathways (Turke et al., 2012; Cheng et al., 2015). We hypothesized that heat stress-induced ENO1 functions *via* the PI3K/Akt pathway. Upregulation ENO1 in the heat stress group significantly elevated Akt mRNA and protein levels. Meanwhile, PI3K and Akt expressions were decreased when ENO1 cells were suppressed. Interestingly, we detected the expression of FAK, an upstream signal factor of the PI3K/Akt pathway, which can promote cell migration and invasion (Nowicki et al., 2011; Yousif, 2014; Fu et al., 2015), and found that upregulation ENO1 during heat stress decreased the mRNA and protein levels of FAK. We conjectured that ENO1 might modulate cell viability and glycolysis *via* the FAK-PI3K/Akt pathway. To further clarify the mechanism, we used Ang II, a FAK accelerant (Dong et al., 2013), and wortmannin, a PI3K/Akt inhibitor (Abliz et al., 2015), to treat ENO1 downregulation and upregulation cells under heat conditions, respectively. We observed that FAK mRNA and protein levels both increased, but there was no significant difference in ENO1 (*p* > 0.05, Figures 6A,C) after Ang II treatment in ENO1-suppressed cells during heat stress.

This may be explained that there is an ENO1-to-FAK pathway but no feedback regulation existing in heat stress. In upregulated ENO1 cells, we found that wortmannin decreased the mRNA and protein levels of ENO1, FAK, PI3K, and Akt. We speculated that upregulated ENO1 increased cell viability and reduced heat stress-induced LDHA release by activating the FAK-mediated PI3K/Akt pathway against heat stress.

## Conclusion

In summary, ENO1 is overexpressed to elevate cell viability and reduced heat stress-induced LDHA release by activating FAK-mediated PI3K/Akt against heat stress. In addition, we provided strong evidence that ENO1 interacts with HSP70 in this process. These data point to new insight into ENO1’s role in heat stress.

## Data Availability Statement

The raw data supporting the conclusions of this article will be made available by the authors, without undue reservation.

## Author Contributions

LL and TZ conceived the experiments and supervised the project. TZ, YC, LC, YT, and GL performed the experiments. TZ, YC, JS, TG, and ZT analyzed and interpreted the data. TZ and YC wrote the manuscript with help from all of the authors. All authors read and approved the final manuscript.

## Conflict of Interest

The authors declare that the research was conducted in the absence of any commercial or financial relationships that could be construed as a potential conflict of interest.
